# Object detection in natural scenes: Independent effects of spatial and category-based attention

**DOI:** 10.3758/s13414-017-1279-8

**Published:** 2017-01-30

**Authors:** Timo Stein, Marius V. Peelen

**Affiliations:** 1 Department of Psychology, University of Amsterdam, Amsterdam, The Netherlands; 2 Center for Mind/Brain Sciences, University of Trento, Trento, Italy

**Keywords:** Natural scenes, Voluntary attention, Visual attention, Expectation, Detection

## Abstract

Humans are remarkably efficient in detecting highly familiar object categories in natural scenes, with evidence suggesting that such object detection can be performed in the (near) absence of attention. Here we systematically explored the influences of both spatial attention and category-based attention on the accuracy of object detection in natural scenes. Manipulating both types of attention additionally allowed for addressing how these factors interact: whether the requirement for spatial attention depends on the extent to which observers are prepared to detect a specific object category—that is, on category-based attention. The results showed that the detection of targets from one category (animals or vehicles) was better than the detection of targets from two categories (animals and vehicles), demonstrating the beneficial effect of category-based attention. This effect did not depend on the semantic congruency of the target object and the background scene, indicating that observers attended to visual features diagnostic of the foreground target objects from the cued category. Importantly, in three experiments the detection of objects in scenes presented in the periphery was significantly impaired when observers simultaneously performed an attentionally demanding task at fixation, showing that spatial attention affects natural scene perception. In all experiments, the effects of category-based attention and spatial attention on object detection performance were additive rather than interactive. Finally, neither spatial nor category-based attention influenced metacognitive ability for object detection performance. These findings demonstrate that efficient object detection in natural scenes is independently facilitated by spatial and category-based attention.

Human observers can rapidly detect the presence of familiar object categories (e.g., animals, vehicles) in photographs of natural scenes (Fabre-Thorpe, 2011; Thorpe, Fize, & Marlot, 1996). Detection in natural scenes is so rapid—evoked potentials differentiate target from nontarget trials within 150 ms (Thorpe et al., 1996; VanRullen & Thorpe, 2001)—that it is thought to reflect feedforward visual processing (Liu, Agam, Madsen, & Kreiman, 2009; Serre, Oliva, & Poggio, 2007; Thorpe & Fabre-Thorpe, 2001). The visual system thus may be capable of detecting objects in natural scenes without attentional feedback.

This notion was tested in a study using a dual-task design (Li et al. 2002). Participants in this study detected animals or vehicles in natural scene images presented in the periphery while, in some conditions, simultaneously performing another highly attention-demanding task at fixation. The results showed that object detection in natural scenes—unlike other visual discrimination tasks—was remarkably unaffected by the attention-demanding task at fixation. The authors concluded that object detection in natural scenes can be performed in the (near) absence of attention (but see Cohen, Alvarez, & Nakayama, 2011, for attention effects in another dual-task setting; see the General Discussion).

In the present study, we investigated the role of attention in object detection in natural scenes in more detail. Our starting point is the distinction between spatial attention and content-based (e.g., feature-based) attention (Carrasco, 2011). Spatial attention refers to the location that is attended while content-based attention refers to the stimulus properties that are attended (Stein & Peelen, 2015)—what an observer is looking for. Previous studies that investigated the relationship between spatial attention and feature-based attention have shown that they are both independent and interactive. They are independent in that feature-based attention modulates visual processing globally across the visual field (Saenz, Buracas, & Boynton, 2002; Serences & Boynton, 2007; Treue & Martínez-Trujillo, 1999). They are interactive in that spatial attention is guided to items that match the feature-based attentional set (Bichot, Rossi, & Desimone, 2005; Wolfe, Cave, & Franzel, 1989).

The concept of content-based attentional templates is also an important aspect of the biased competition model of attention (Desimone & Duncan, 1995; Duncan & Humphreys, 1989). According to this model, attentional templates bias the processing of incoming visual input in favor of currently relevant stimuli. Importantly, attentional templates are not restricted to one type of visual property, such as a target’s low-level features, but may also include more complex features encoded at higher stages of the visual processing hierarchy when these properties best distinguish targets from nontargets (Peelen & Kastner, 2014).

Previous studies investigating the role of content-based attentional templates in the detection of familiar object categories in natural scenes have confirmed that attention can be directed not just at the level of simple features but also at the level of object category. Although it is not fully known what features such category-based attentional templates consist of, they likely include bundles of category-diagnostic features of intermediate complexity (Crouzet & Serre, 2011; Delorme, Richard, & Fabre-Thorpe, 2010; Evans & Treisman, 2005; Reeder & Peelen, 2013; Ullman, Vidal-Naquet, & Sali, 2002). Interestingly, similar to the effects of feature-based attention, when observers search for a particular object category, spatial attention is captured by exemplars of this category at task-irrelevant locations, indicating a spatially global effect of category-based attention (Reeder & Peelen, 2013; Reeder, van Zoest, & Peelen, 2015). Spatially global effects of category-based attention were also observed in neural responses in visual cortex, with category-based attention modulating visual processing of both spatially attended and spatially unattended scenes (Peelen, Li, & Kastner, 2009).

Considering these findings, we hypothesized that efficient detection in natural scenes outside the focus of spatial attention may critically depend on category-based attention: When observers prepare to detect targets of the task-relevant object category, the activation of a categorical attentional template allows for the efficient processing of visual input that matches the template (i.e., targets). Because attentional templates are thought to represent one object at a time (Olivers et al., 2011), category-based attention is fully available only when preparing for one specific target object (rather than for two or more potential target objects simultaneously). Therefore, we hypothesized that rapid object detection may be performed in the absence of spatial attention but only when observers detect one category at a time. This result would demonstrate that feedforward perception of natural scenes critically depends on the attentional state of the visual system at the moment of scene onset.

To test these hypotheses, we proceeded as follows: In Experiment 1, we measured the influence of category-based attention on object detection in natural scenes. In this experiment, spatial attention was always fully available. In Experiment 2, we validated a dual-task procedure for manipulating spatial attention, using artificial stimuli. Finally, Experiment 3 adopted this dual-task procedure to measure the interplay of spatial attention and category-based attention in natural scene perception.

## Experiment 1

**Fig. 1:**
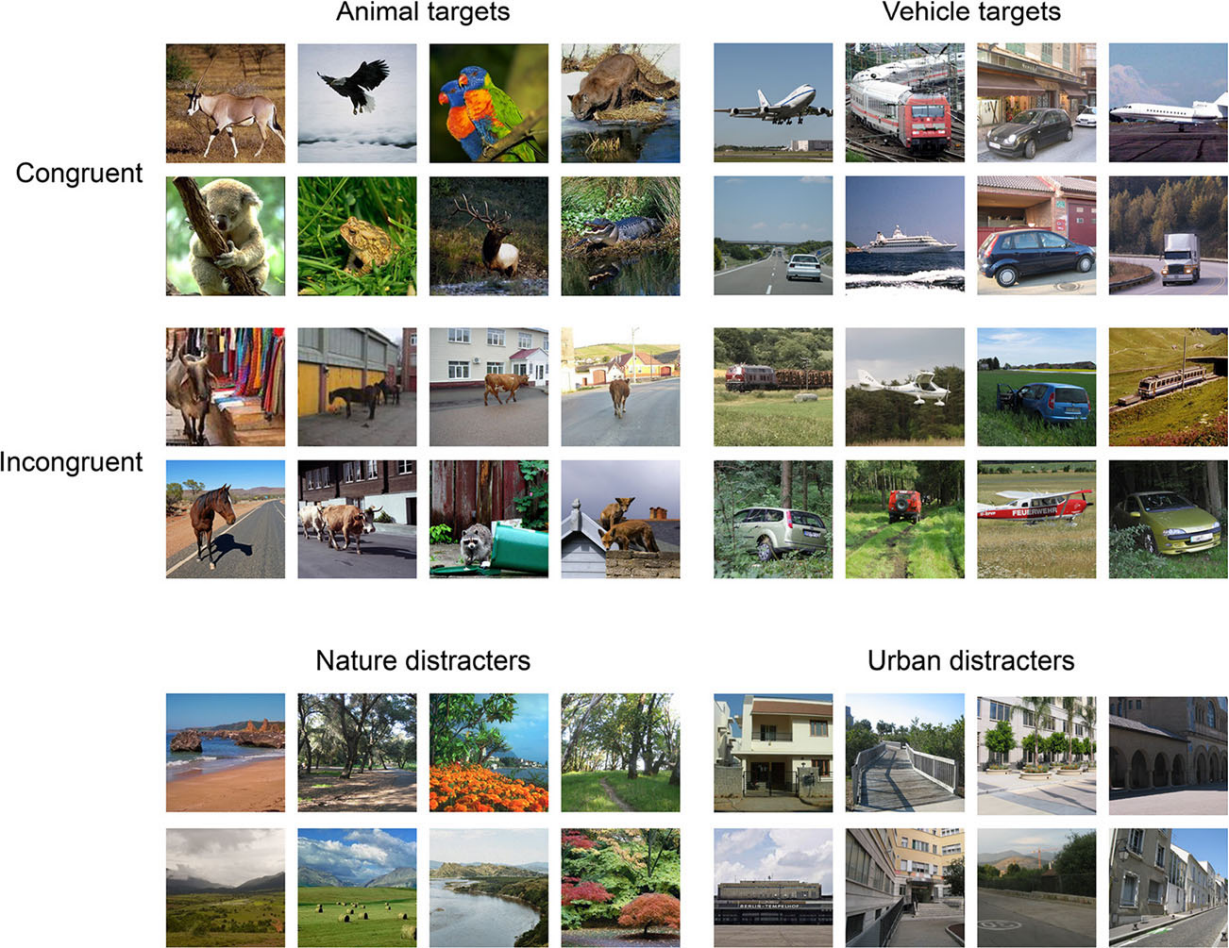
Example stimuli used in Experiments 1, 3a, and 3b

Before studying the interplay of category-based and spatial attention during object detection in natural scenes, in Experiment 1 we first examined the influence of category-based attention on the detection of animal and vehicle targets. To our knowledge, no previous study has directly tested whether category-specific preparation improves object detection at the superordinate level (e.g., animal/vehicle detection). One recent study demonstrated the beneficial influence of prior information about scene gist (e.g., beach, mountain) on categorizing rapid streams of images as either containing or not containing the cued gist category (Evans, Horowitz, & Wolfe, 2011). In our own previous work, we found that prior information about basic-level object categories (e.g., cat, guitar) improved simple detection performance for objects from the cued categories (Stein & Peelen, 2015; see also Lupyan & Ward, 2013; Pinto, van Gaal, de Lange, Lamme, & Seth, 2015). In Experiment 1, we tested whether this beneficial influence of top-down preparation extended to the detection of superordinate categories in natural scenes. We hypothesized that prior information about the category of the target that was provided by a word cue (animal or vehicle) would improve detection performance. An auxiliary question was whether this putative cueing effect would depend on the typicality of the scene photograph. In the congruent condition, animals and vehicles were presented in a typical scene background, such as urban scenes for vehicles and nature-related scenes for animals. In the incongruent condition, animal and vehicle targets were embedded in a less typical background, such as nature-related scenes for vehicles and urban scenes for animals (see Fig. 1). If observers prepared for the overall gist or overall dominating colors of the scenes typically associated with vehicles and animals, respectively, cueing effects would be expected to be larger for congruent scenes. Alternatively, if observers more specifically prepared for the visual features of the foreground target object category, cueing effects would be similar for congruent and incongruent scenes.

### Method

#### Participants

For all experiments, volunteers were recruited through the University of Trento subject pool. They participated for course credit or payment. All participants reported normal or corrected-to-normal vision and were naïve as to the purposes of the experiments. Eighteen participants took part in Experiment 1a (15 female, three male; age range 19–27 years, mean 22.7 years). In Experiment 1b, 18 participants also took part (15 female, three male; age range 19–27 years, mean 22.4 years), of whom 14 had participated in Experiment 1a.

#### Apparatus and stimuli

The stimuli were presented on a 19-in. CRT monitor (1,024 × 768 pixels resolution, 60-Hz refresh rate) with MATLAB (The MathWorks, Natick, MA) using the Cogent 2000 toolbox (www.vislab.ucl.ac.uk/cogent.php) and the Psychophysics Toolbox (Brainard, 1997) functions. The observer’s head was stabilized by a chin-and-head rest at a viewing distance of approximately 50 cm. A gray square (32 cd/m^2^, visual angle of approx. 5.0° × 5.0°) was centered on the black background throughout the experiments. A black fixation cross, word cues (Arial font, 22 points), scene photographs (5.0° × 5.0°), and masks (5.0° × 5.0°) were centered in this square.

Color photographs of real-world scenes were gathered from various sources, including Google image search and the LabelMe online database (Russell, Torralba, Murphy, & Freeman, 2008). The 160 animal target scenes included mammals, birds, and reptiles, and the 160 vehicle target scenes included cars, trucks, trains, airplanes, and ships. Half of the target images within each category were “congruent,” meaning that the target object was shown in a typical scene background (e.g., antelope in the savannah, car in a street scene), and half of the target images within each category were “incongruent,” meaning that the target object was shown in a less-common scene background (e.g., horse on the highway, airplane on a field; see Fig. 1 for some examples). In most cases, the background of incongruent scenes from one target category was a common scene background of congruent scenes from the other target category. That is, most incongruent animal targets were shown in urban backgrounds, and most incongruent vehicle targets were shown in nature-related scenes. The nontarget distracter scenes were 1,100 images of a wide range of urban and nature-related scenes not containing animals or vehicles. Finally, masks were sampled from 576 color images consisting of a mixture of noise at different spatial frequencies with superimposed naturalistic textures (e.g., Peelen et al., 2009; Walther, Caddigan, Li, & Beck, 2009).

#### Procedure

**Fig. 2:**
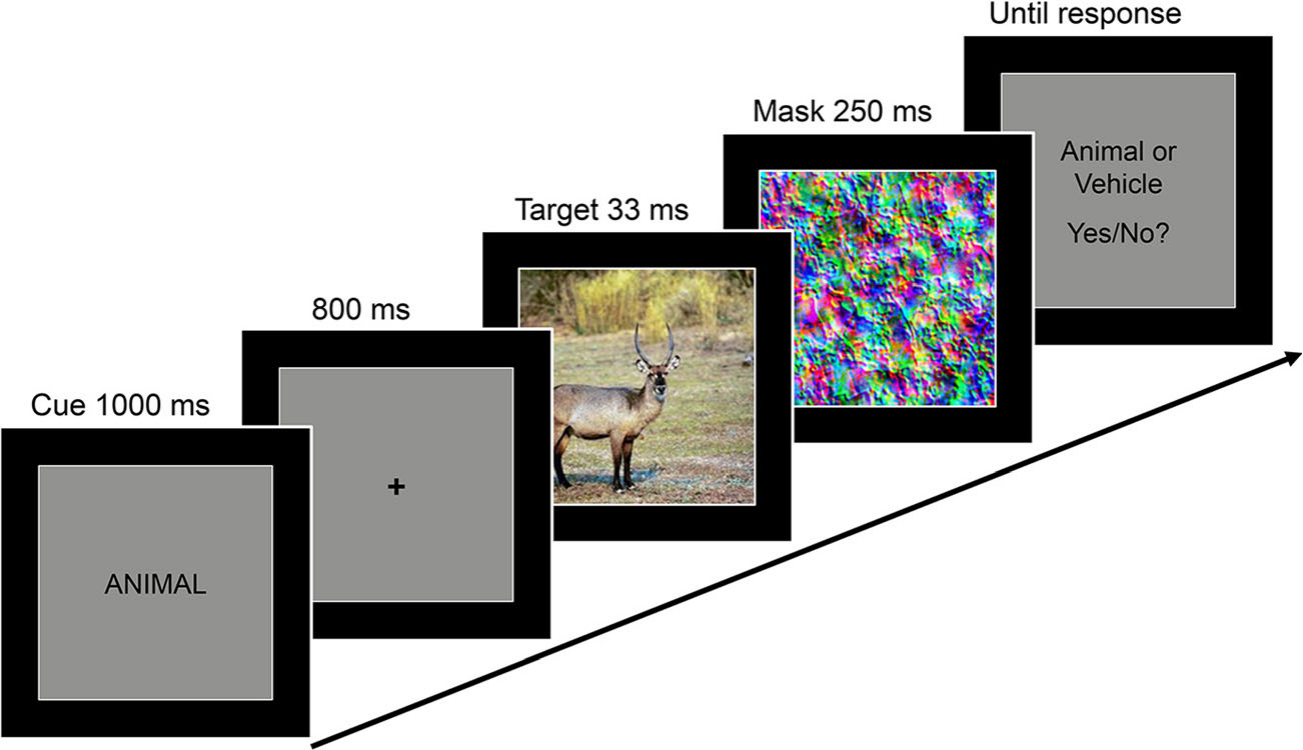
Schematic of an example trial from Experiment 1. At the beginning of a trial, a word provided either no information, valid information, or invalid information about the relevant category for the upcoming detection task. A scene was then presented briefly and immediately masked. For all cue conditions, participants performed the same present–absent detection task, indicating whether the scene contained either an animal or a vehicle (“yes” response) or neither of these categories (“no” response)

A schematic trial sequence is depicted in Fig. 2. Each trial began with a 300-ms presentation of the gray square only, which was followed by a 1-s presentation of a word cue.

In Experiment 1a, the word was either “READY” (noncued condition, 50% of all trials) or the Italian word corresponding to the relevant category for the upcoming detection task—that is, either “ANIMALE,” for animal detection, or “VEICOLO,” for vehicle detection (cued condition, 50% of all trials). Importantly, in all trials (irrespective of the cue condition), participants had to decide whether the scene contained an animal or a vehicle (and respond “yes”), or whether it contained neither of these categories (and respond “no”). As such, participants could also perform the task perfectly by completely ignoring the word cues. The word cues always validly indicated the target category, if it was present in the scene. These task requirements were explained through verbal and written instructions.

In Experiment 1b, the word cue could now either validly or invalidly predict the target category. In valid trials (75% of the target-present trials), the word corresponded to the upcoming target category, while in invalid trials (25% of the target-present trials), the word corresponded to the other target category. In target-absent trials, one of the category words was randomly drawn, with the constraint that both category words occurred with equal probabilities. Participants were informed that these word cues correctly predicted the target category in most, but not all, trials. They were instructed that they always needed to decide whether the scene contained an animal or vehicle, or neither of these categories. In both experiments, participants were asked to pay attention to (i.e., to read) the cues.

The presentation of the cue was followed by an 800-ms fixation period. A scene image was then presented for 33 ms, followed immediately by the presentation of a mask, which remained on the screen for 250 ms. Participants were then required to indicate as accurately as possible, without speed pressure, whether or not a target had been presented, using the left and right arrow keys on the keyboard. They were informed that two thirds of the trials contained a target and that the targets could be either animals or vehicles. Twelve practice trials in which the target was presented for a longer duration (66 ms) preceded the experiment proper, to familiarize participants with the cue–target–response sequence. After entering their response, participants received feedback (the fixation cross turned either green or red).

Each experiment contained 480 trials (separated by obligatory breaks after 120, 240, and 360 trials), with 320 target-present and 160 target-absent trials. In Experiment 1a, in the target-present trials each combination of two cue conditions (cued, noncued), two target categories (animals, vehicles), and target–background congruency (congruent, incongruent) was presented equally often. The same 160 target scenes were presented in the cued and the noncued conditions. In Experiment 1b, in the target-present trials there were 240 valid and 80 invalid trials, in which each combination of two target categories and target–background congruency occurred equally often. The target scenes were sampled randomly without replacement (i.e., different target scenes were presented in the valid and the invalid conditions). In both experiments, in the target-absent trials distracter scenes were randomly sampled (without replacement) from a set of 1,100 images. Trial order was randomized.

#### Analysis

Our central question was whether detection sensitivity would differ as a function of prior knowledge about the relevant category in the upcoming detection task. In addition, we tested whether this cueing effect would be affected by target–background congruency. For Experiment 1a, the hit rates, computed separately for the four combinations of cue and congruency conditions, and false alarm rates, computed separately for the two cue conditions (there was no congruency manipulation in target-absent scenes), were *z*-transformed and converted to the sensitivity measure *d'*, applying the log-linear correction by Hautus (1995). For Experiment 1b, we analyzed the hit rates computed for the four combinations of cue and congruency conditions in target-present trials, because there was no manipulation of cue validity in target-absent trials.

### Results and discussion

**Fig. 3:**
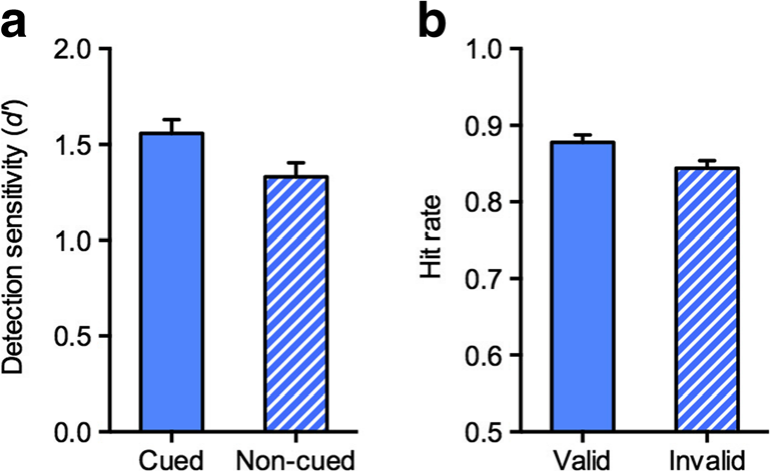
Results from (**a**) Experiment 1a and (**b**) Experiment 1b. *Error bars* represent the *SE*s of the differences between the cued and noncued conditions (Exp. 1a) and between the valid and invalid conditions (Exp. 1b), respectively

A repeated measures analysis of variance (ANOVA) with the factors Cue (cued, noncued) and Target–Background Congruency (congruent, incongruent) on the *d'* scores from Experiment 1a revealed a significant main effect of cue, *F*(1, 17) = 10.54, *p* = .005, *η*_p_^2^ = .38, with higher sensitivity for cued targets (*M =* 1.54) than for noncued targets (*M* = 1.31; see Fig. 3a); a significant main effect of congruency, *F*(1, 17) = 16.91, *p* = .001, *η*_p_^2^ = .50, with higher sensitivity for congruent scenes (*M =* 1.51) than for incongruent scenes (*M* = 1.34), consistent with better perception of probable than of improbable scenes (Greene, Botros, Beck, & Li, 2015); but no significant interaction, *F*(1, 17) = 2.21, *p* = .156, *η*_p_^2^ = .12. Thus, advance knowledge of the target category increased detection sensitivity. Interestingly, this cueing effect did not differ between congruent scenes, in which the target object was embedded in a typical background, and incongruent scenes, in which the target object was embedded in a less-common scene background. This indicates that participants were not simply preparing for the gist or for the overall dominating colors commonly associated with animal and vehicle scenes, respectively. Rather, it seems that observers prepared for visual features diagnostic of foreground target objects from the cued category.

To ensure that this advantage did not reflect a nonspecific effect of the word cue denoting the task-relevant object category, as compared to the noninformative “READY” cue (e.g., differences in alertness), in Experiment 1b we compared detection performance in trials with valid and invalid category cues. Also in this experiment, we found a significant cueing effect, *F*(1, 17) = 12.03, *p* = .003, *η*_p_^2^ = .41, with higher hit rates for validly cued targets (*M* = .88) than for invalidly cued targets (*M* = .84; see Fig. 3b), but no significant main effect of congruency, *F* < 1, and no significant interaction, *F*(1, 17) = 3.20, *p* = .092, *η*_p_^2^ = .16. The absence of a main effect of congruency might have reflected greater familiarity with the incongruent scenes than in Experiment 1a, since most of the participants from Experiment 1b had taken part in Experiment 1a first. More importantly, the significant cueing effect demonstrates that detectability was enhanced only when the target matched the category indicated by the word cue. Thus, the results from Experiment 1 demonstrate that category-based attention can enhance the detection of target objects from superordinate categories in natural scenes.

## Experiment 2

Whereas in Experiment 1 the scenes were always spatially attended, in the following experiments we additionally manipulated spatial attention through a dual-task procedure. In Experiment 2, we first established the effectiveness of our spatial attention manipulation. For this, we measured the influence of the central fixation-dimming task on the discrimination of color patterns in the periphery, a task that is known to require spatial attention (Lee, Koch, & Braun, 1999; Li et al., 2002).

### Method

#### Participants

Nine participants took part in Experiment 2 (six female, three male; age range 20–34 years, mean 26.7 years).

#### Stimuli

**Fig. 4:**
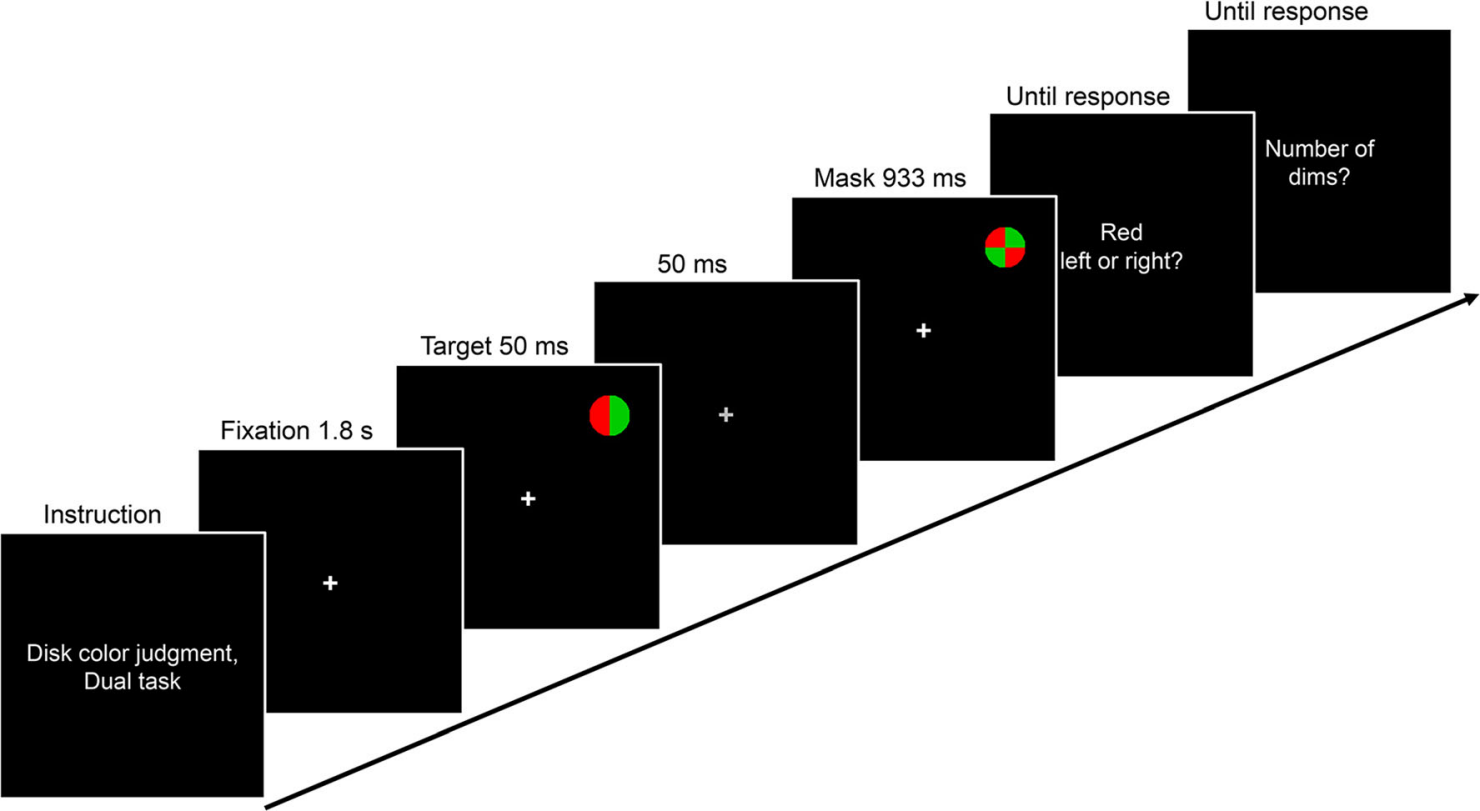
Schematic of a dual-task example trial from Experiment 2. On every trial, a red–green colored disk was briefly presented in one of four positions in the periphery, followed by a mask. Concurrently, the luminance of the fixation cross was dimmed between one and four times during each trial. In different blocks, participants were required to count the number of fixation dims (central single task), to discriminate the color pattern of the disk (peripheral single task), or to perform both tasks (dual task)

The general setup was identical to that of Experiment 1, but stimuli were now presented against a black background, and the fixation cross and all text were white. The targets were red–green or green–red colored disks and the mask was a red–green checkered disk (2.0° × 2.0°; cf. Li et al., 2002, and see Fig. 4).

#### Procedure

Experimental conditions were run in separate blocks to minimize potential confusion due to continuous task switching. Participants received detailed verbal and written instructions and at least five practice trials at the beginning of each of the three parts of the experiment. Three different experimental conditions were presented in a fixed sequence: peripheral color discrimination single task, central fixation-dimming single task, and dual task.

All participants first completed two blocks of the peripheral color discrimination single task. The general trial layout for the dual task is illustrated in Fig. 4. Each trial began with an 800-ms presentation of a blank screen, followed by the 1.8-s presentation of the white fixation cross only. The target disk was then displayed for 50 ms in one of the quadrants (centered at an eccentricity of 6.7°), followed by a 50-ms blank (fixation only), and finally by the onset of the mask, which remained on the screen for 933 ms. Participants were required to indicate as accurately as possible, without speed pressure, whether the disk was colored red–green or green–red, using the left and right arrow keys on the keyboard. They were informed that both targets were equally likely. After entering their response, participants received feedback (the fixation cross turned either green or red). Each block contained 80 trials, in which each combination of two target disks and the four quadrants for target presentation occurred ten times.

Next, participants completed five practice trials and one block of the central fixation-dimming single task. The trials were similar, except that no disk target and no mask were presented, and that the fixation cross was dimmed (from approximately 63 cd/m^2^ to approximately 49 cd/m^2^) for 67 ms between one and four times per trial. The dims could occur at any time from 1.6 s after trial onset until the end of the stimulus presentation sequence (3.6 s after trial onset). The number and temporal position of the fixation dims was determined at random for each trial, with the constraint that two consecutive dims were separated by at least 300 ms. At the end of the trial, participants were required to indicate as accurately as possible, without speed pressure, the number of dims they had counted, using the number keys on the keyboard. They received feedback. The block contained 80 trials.

Finally, participants performed two blocks of the dual task, which combined the peripheral color discrimination single task and the central fixation-dimming single task. The instructions emphasized that both tasks would be equally important, that participants should try to perform as well as possible in both tasks, and should focus their attention at the center of the screen. At the end of each trial, participants first responded to the peripheral color discrimination task and then to the central fixation-dimming task. Each block contained 80 trials.

#### Analysis

Performance on the central fixation-dimming task was computed as a proportion correct, separately for the single- and dual-task conditions. Sensitivity in peripheral color discrimination was determined by converting hit rates (i.e., red–green judgments for red–green disks) and false alarm rates (i.e., red–green judgments for green–red disks) to *d'* scores, separately for the single- and dual-task conditions.

### Results and discussion

**Fig. 5:**
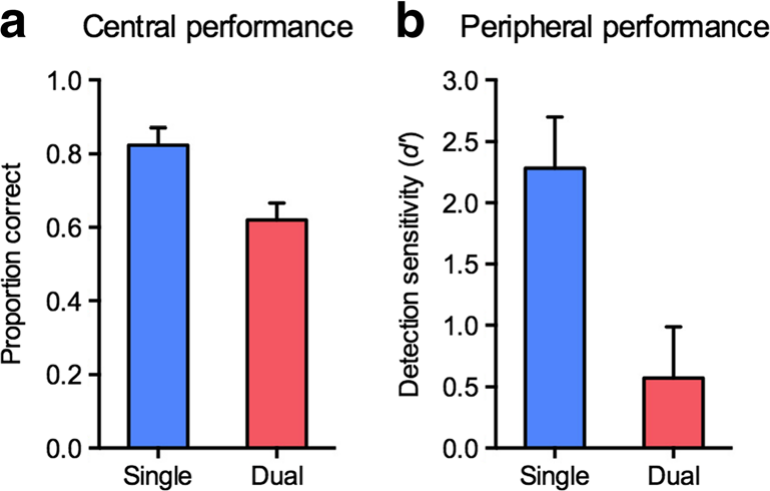
Results from Experiment2. (**a**) Mean proportions correct for counting the number of central fixation dims, shown separately for the single- and the dual-task conditions. (**b**) Mean *d'* scores for discriminating the color pattern of the peripherally presented disk, shown separately for the single- and dual-task conditions. *Error bars* represent the *SE*s of the differences between the single- and the dual-task conditions.

Experiment 2 was conducted to determine whether the central fixation-dimming task was effective in drawing spatial attention. As can be seen in Fig. 5a, accuracy in counting the number of fixation dims was significantly higher in the single-task (*M* = .82) than in the dual-task (*M* = .61) condition, *t*(8) = 4.69, *p* = .002, *d* = 1.56. Also, *d'* scores for the peripheral color discrimination were significantly higher in the single-task (*M* = 2.28) than in the dual-task (*M* = 0.57) condition, *t*(8) = 4.14, *p* = .003, *d* = 1.38 (see Fig. 5b). Thus, performance in the color discrimination task, which is known to be strongly dependent on spatial attention (Lee et al., 1999; Li et al., 2002), decreased considerably when participants also had to count fixation dims. Note that all participants underwent the single tasks before the dual task, such that potential practice effects should have led to a smaller rather than a larger decrement in performance from the single to the dual tasks. The results of Experiment 2 thus demonstrate that the fixation-dimming task was highly effective in drawing spatial attention.

## Experiment 3

Next, we systematically investigated how spatial attention interacts with category-based attention in natural scene perception. Spatial attention was either available for the detection of objects in natural scenes in the periphery (single task) or drawn away by the concurrent central fixation-dimming task (dual task).

In Experiment 3a, we first tested the influence of spatial attention on the detection of one precued target category (animals or vehicles, cued condition) versus the detection of two categories (animals and vehicles, noncued condition) in peripherally presented natural scenes.

Experiment 3b was designed to provide an independent replication and to explore how spatial and category-based attention affect introspective or metacognitive ability. *Metacognitive ability* refers to the relationship between objective performance measures, such as object detection performance, and subjective confidence in the accuracy of this response (Galvin, Podd, Drga, & Whitmore, 2003; Kunimoto, Miller, & Pashler, 2001; Song et al., 2011). Metacognition is often taken as an index of subjective awareness, as opposed to performance-based objective awareness measures (Kunimoto et al., 2001; Szczepanowski & Pessoa, 2007; Wilimzig, Tsuchiya, Fahle, Einhäuser, & Koch, 2008). Previous work indicated that spatial attention and expectations may have dissociable effects on metacognitive ability: Whereas manipulations of spatial attention did not influence metacognition in tasks requiring the detection and discrimination of oriented gratings (Sherman, Seth, Barrett, & Kanai, 2015; Wilimzig et al., 2008), improved metacognition has been found when present/absent reports matched the expected probabilities of present/absent trials in a grating detection task (Sherman et al., 2015). In Experiment 3b, we tested whether a similar dissociation would be found between the effects of spatial and category-based attention on metacognitive ability in natural scene perception.

Finally, in Experiment 3c we sought to replicate our findings with another stimulus set. Instead of the specific set of congruent and incongruent images included in the previous experiments, for Experiment 3c we used a set of scene photographs that has been used in previous studies on object detection in natural scene (e.g., Li et al., 2002; Thorpe et al., 1996).

### Method

#### Participants

In Experiment 3a, there were 14 participants (eight female, six male; age range 19–28 years, mean 23.6 years), of whom two had participated in Experiment 2 (on a separate day). Eleven participants took part in Experiment 3b (ten female, one male; age range 19–53 years, mean 29.5 years). In Experiment 3c, we recruited 12 participants; one of these participants was excluded from the analysis because his detection sensitivity was close to chance level (across all conditions, *d'* of 0.03). Of the remaining 11 participants (eight female, three male; age range 19–53 years, mean 25.5 years), one participant had taken part in Experiment 3a, and another had taken part in Experiment 3b (in separate sessions).

#### Stimuli

Experiment 3 was the same as Experiment 2, except that scene photographs were presented at the periphery. In Experiments 3a and 3b, we used a subset of the scene photographs from Experiment 1, resulting in a set of 40 animal target scenes (20 congruent, 20 incongruent), 40 vehicle target scenes (20 congruent, 20 incongruent), and 1,100 randomly sampled nontarget distracter scenes. Scene photographs and masks were scaled to 8.3° × 8.3° and centered at an eccentricity of 11.2° in one of the quadrants. For the practice blocks, another subset of scene photographs from Experiment 1 was used. In Experiment 3c, we used another stimulus set: Scene photographs were randomly sampled from a commercially available library of color scene photographs, containing more than 500 images of animals, vehicles, and nontarget distracter scenes (Li et al., 2002; Thorpe et al., 1996). Scene photographs and masks were scaled to 12.0° × 8.0° and centered at an eccentricity of 12.4° in one of the quadrants.

#### Procedure

Seven different tasks were presented in separate blocks to avoid confusion. Participants received detailed verbal and written instructions at the beginning of each part of the experiment. The experimental session started with one block of 80 trials of the central fixation-dimming single task, identical to that in Experiment 2.

Next, participants practiced the peripheral object detection single task. One block of the animal detection single task, one block of the vehicle detection single task, and two blocks of the animal-and-vehicle detection single task were presented in a random sequence. Note that these peripheral object detection tasks followed the same logic as the central object detection tasks in Experiment 1: Nontarget trials in the animal detection task never contained vehicles, and nontarget trials in the vehicle detection task never contained animals. This was followed by four practice blocks of the dual task, in which participants performed both the central fixation-dimming and the peripheral object detection task. One block of the animal detection dual task, one block of the vehicle detection dual task, and two blocks of the animal-and-vehicle detection dual task were presented in random order. Each practice block contained 24 trials.

**Fig. 6:**
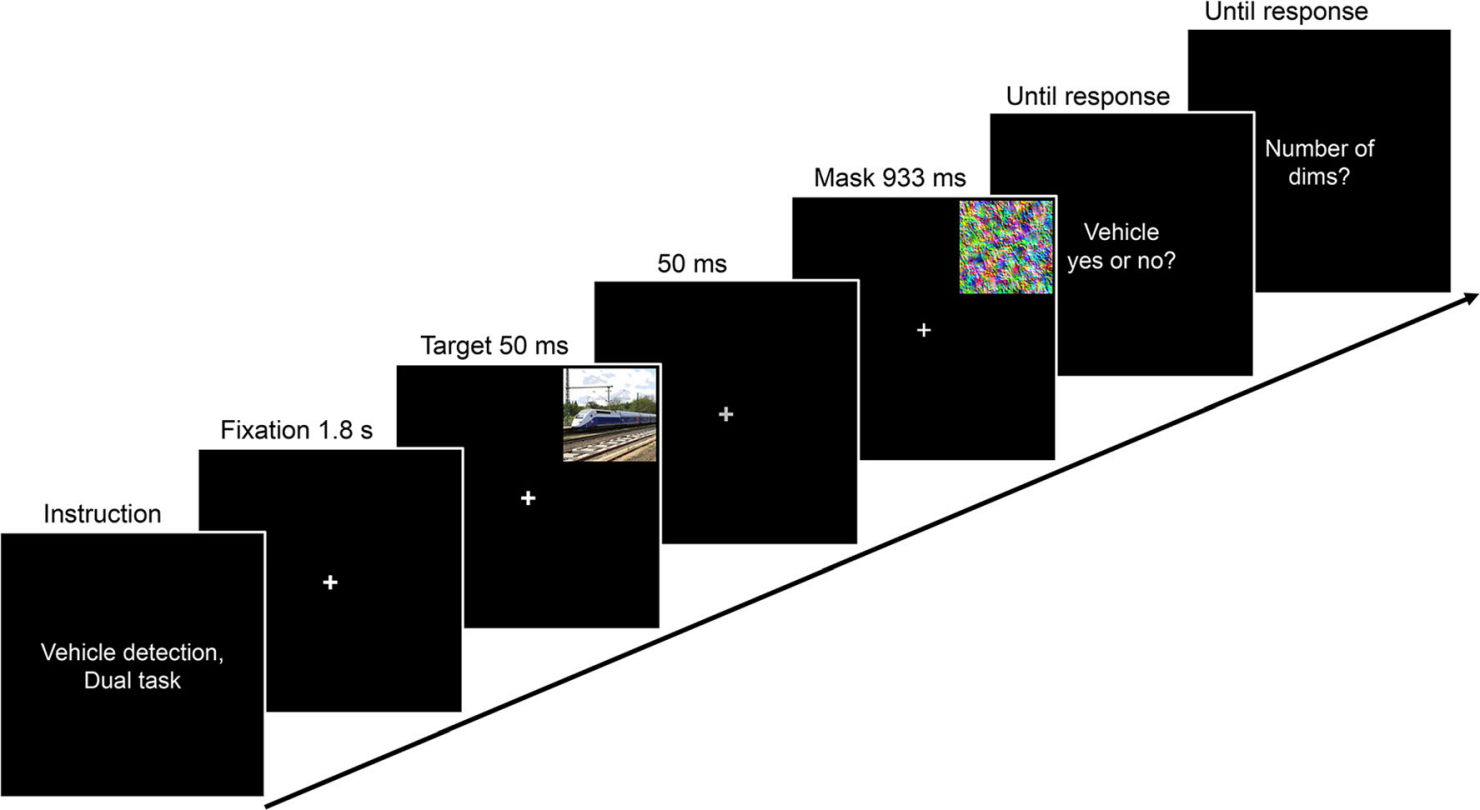
Schematic of a dual-task example trial from Experiment 3a. On every trial, a scene photograph was presented briefly in one of four positions in the periphery, followed by a mask. Concurrently, the luminance of the fixation cross was dimmed between one and four times during each trial. In different blocks, participants were required to count the number of fixation dims (central single task), to detect animals and vehicles in the scenes (peripheral single task), or to perform both tasks (dual task). Importantly, before every block, participants were instructed whether they needed to detect animals only or vehicles only (cued conditions), or whether they needed to detect both animals and vehicles (noncued condition)

In the following experiment proper, 16 blocks of 40 trials each were presented in a random sequence: eight blocks of the single tasks (two blocks animal detection single task, two blocks vehicle detection single task, and four blocks animal-and-vehicle detection single task) and eight blocks of the dual tasks (two blocks animal detection dual task, two blocks vehicle detection dual task, and four blocks animal-and-vehicle detection dual task). At the beginning of each block, participants received instructions (e.g., “Vehicle detection, dual task” or “Animal and vehicle detection, single task”; see Fig. 6).

An example trial of the vehicle detection dual task is illustrated in Fig. 6. The trial structure was similar to that of Experiment 2, except that scene photographs replaced the disk targets, and noise masks (identical to those used in Exp. 1) replaced the checkered-disk masks. Across blocks, the same animal and vehicle target scenes were presented in the single- and dual-task conditions. Also, in blocks in which participants were required to detect targets from one precued target category (animals or vehicles, cued condition) and blocks in which they needed to detect targets from two categories (animals and vehicles, noncued condition), the same animal and vehicle target scenes were used. At the end of the stimulus presentation sequence, participants were required to indicate as accurately as possible, without speed pressure, whether or not a target had been presented, using the left and right arrow keys on the keyboard. They were informed that half of the trials contained a target. In dual-task blocks, participants additionally performed the fixation-dimming task, which was identical to that in Experiment 2. In a block of 40 trials, 20 target-present and 20 target-absent trials were presented, and each quadrant for scene presentation occurred ten times.

In Experiments 3a and 3c, participants received feedback after responding to the peripheral object detection task. In Experiment 3b, in which we measured metacognitive ability, participants received no feedback after entering their response to the peripheral object detection task, but instead rated their confidence on a scale from 1 to 4, with 1 representing *low* and 4 representing *high* confidence in the participant’s own present–absent response. Participants were asked to use the whole scale. They were also instructed that the scale reflected relative confidence, because they might never be fully confident within the context of the perceptually demanding task.

#### Analysis

Performance on the central fixation-dimming task was computed as the proportion correct, separately for the single-task conditions and the dual-task cued and noncued conditions. For the peripheral object detection task, *d'* scores were computed from the differences between the *z*-transformed hit and false alarm rates separately for the four possible combinations of single- and dual-task conditions and cued and noncued conditions. For the cued condition, data from the animal and vehicle detection blocks were collapsed. Because Experiment 1 did not reveal significant interactions with scene-background congruency, here we did not consider this factor anymore (exploratory analyses of Experiment 3a again revealed no significant interactions with congruency, all *p*s > .226, and no significant main effect of congruency, *p* = .203).

For Experiment 3b, metacognitive performance was computed as the relationship between the accuracy of the object detection response and subjective confidence in this response using the Type II receiver operating characteristic (ROC) curve (Galvin et al., 2003). We used the same method described in Song et al. (2011). In brief, the ROC curve had three inflection points from the confidence data, where correct responses with higher confidence were regarded as hits and incorrect responses with higher confidence as false alarms. In this analysis, the area under the curve (AUC: area under the Type II ROC curve plus the diagonal) represented metacognitive performance for peripheral object detection. Metacognitive ability was calculated separately for the single-task versus dual-task blocks, and for blocks in which participants detected animals only or vehicles only (cued conditions) versus blocks in which they detected both animals and vehicles (noncued condition). Note that this standard measure of metacognition was calculated on the basis of all trials, including target-present and target-absent responses. Because there is evidence that experimental manipulations can differentially influence metacognition in trials with target-present responses and trials with target-absent responses (Kanai, Walsh, & Tsong, 2010; Meuwese, van Loon, Lamme, & Fahrenfort, 2014), we also calculated metacognitive ability separately for trials with target-absent responses (metacognitive ability for discriminating between correct rejections and misses) and for trials with target-present responses (metacognitive ability for discriminating between hits and false alarms).

### Results and discussion

#### Experiment 3a

**Fig. 7:**
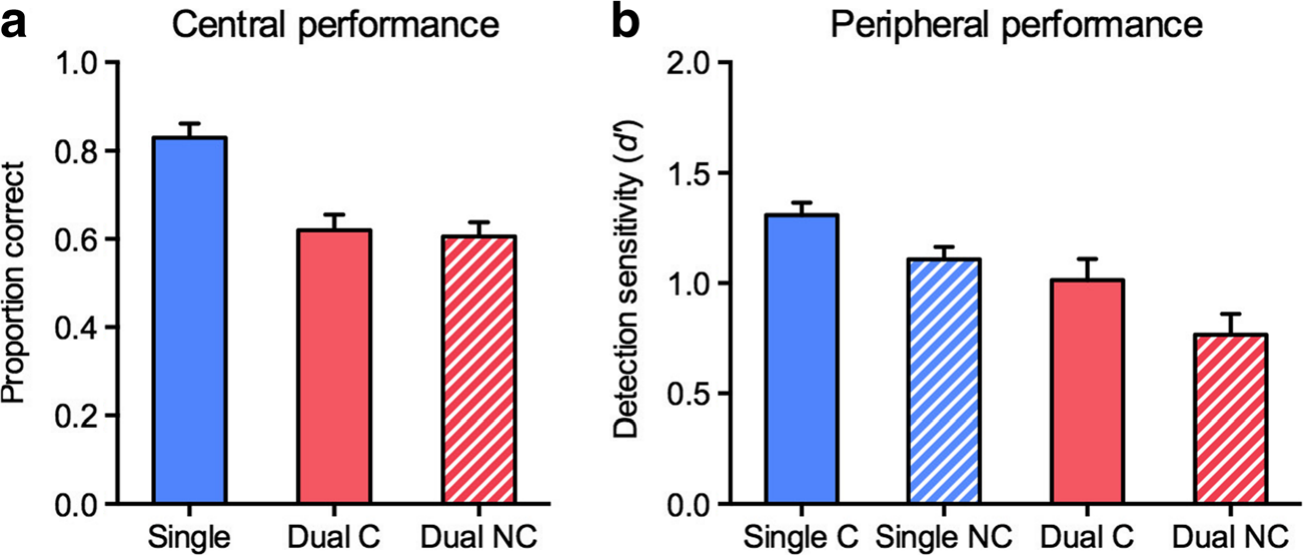
Results from Experiment 3a. (**a**) Mean proportions correct for counting the numbers of central fixation dims, shown separately for the single-task condition, the dual-task cued condition (“Dual C”), and the dual-task noncued condition (“Dual NC”). *Error bars* represent the *SE*s of the differences between the single-task and dual-task conditions. (**b**) Mean *d'* scores for object detection in peripheral scenes, shown separately for the single-task cued (“Single C”), the single-task noncued (“Single NC”), the dual-task cued (“Dual C”), and the dual-task noncued (“Dual NC”) conditions. *Error bars* represent the *SE*s of the differences between the cued and noncued conditions

Accuracy in the central fixation-dimming task was higher in the single-task (*M* = .83) than in the dual-task (*M* = .61) conditions, *t*(13) = 6.68, *p* < .001, *d* = 1.79 (see Fig. 7a). Importantly, accuracy in this central task did not differ between the cued and noncued conditions (*M* = .62, and *M* = .61, respectively; *t* < 1), ruling out differential trade-offs between the central and peripheral tasks in the cued and noncued conditions. Detection sensitivities from the object detection task were analyzed with a repeated measures ANOVA with the factors Cue (cued, noncued) and Task (single, dual). This analysis yielded a significant main effect of cue, *F*(1, 13) = 16.34, *p* = .001, *η*_p_^2^ = .56, with higher sensitivity in the cued condition (*M =* 1.16) than in the noncued condition (*M* = 0.94); a significant main effect of task, *F*(1, 13) = 23.54, *p* < .001, *η*_p_^2^ = .64, with higher sensitivity in the single task (*M =* 1.21) than in the dual task (*M* = 0.89); but no significant interaction, *F*(1, 13) = 0.20, *p* = .664, *η*_p_^2^ = .02. Thus, both spatial attention and advance knowledge of the relevant target category improved object detection in peripheral scenes. Importantly, the influence of category-based attention was independent of spatial attention: As can be seen in Fig. 7b, detection sensitivities were significantly higher in the cued than in the noncued condition in both the single-task, *t*(13) = 3.55, *p* = .004, *d* = 0.95, and the dual-task, *t*(13) = 2.64, *p* = .020, *d* = 0.71, conditions. Also, spatial attention enhanced detection in a way that was independent of category-based attention, with detection sensitivities being higher in single-task than in dual-task conditions, both in the cued, *t*(13) = 3.34, *p* = .005, *d* = 0.89, and in the noncued, *t*(13) = 4.17, *p* = .001, *d* = 1.11, conditions. Thus, spatial attention and category-based attention exerted mutually independent influences on the detection of objects from superordinate categories in natural scenes.

Finally, we examined whether the influence of spatial attention differed between the color discrimination Experiment 2 and the category detection Experiment 3a. This was done to test whether object detection in natural scenes would be less dependent on spatial attention than would a task with less naturalistic, more arbitrary stimuli, such as color pattern discrimination, as had been reported previously (Li et al., 2002). A mixed ANOVA with the between-subjects factors Experiment (2, 3a) and Task (single, dual) yielded a significant interaction, *F*(1, 21) = 17.00, *p* < .001, *η*_p_^2^ = .45: The drop in performance from the single- to the dual-task condition was much larger for the color pattern discrimination task in Experiment 2 (*M* = 1.71) than for the object detection task in Experiment 3a (*M* = 0.32). Another ANOVA with the same factors on central task performance revealed no interaction, *F* < 1. Thus, drawing spatial attention to the central fixation-dimming task had a stronger influence on color pattern discrimination, thus supporting the notion that the detection of familiar object categories in natural scenes is less dependent on spatial attention than are tasks with more artificial stimuli (Li et al., 2002).

#### Experiment 3b

**Fig. 8:**
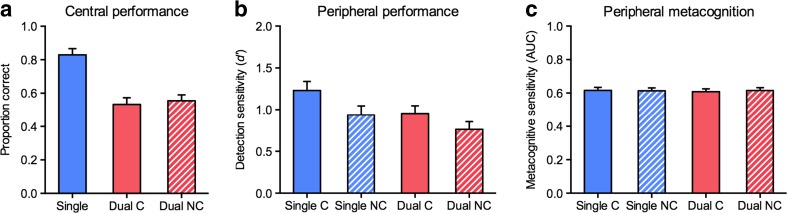
Results from Experiment 3b. (**a**) Mean proportions correct for counting the numbers of central fixation dims, shown separately for the single-task condition, the dual-task cued condition (“Dual C”), and the dual-task noncued condition (“Dual NC”). *Error bars* represent the *SE*s of the differences between the single-task and dual-task conditions. (**b**) Mean *d'* scores for object detection in peripheral scenes, shown separately for the single-task cued (“Single C”), the single-task noncued (“Single NC”), the dual-task cued (“Dual C”), and the dual-task noncued (“Dual NC”) conditions. *Error bars* represent the *SE*s of the differences between the cued and noncued conditions. (**c**) Mean values for the area under the curve (AUC; area under the Type II ROC curve constructed by relating confidence to accuracy, plus the diagonal), representing participants’ metacognitive performance in object detection. Abbreviations and *error bars* are as in panel b

We first analyzed the objective performance measures, as a direct replication of Experiment 3a. Also in Experiment 3b, accuracy in the central fixation-dimming task was higher in the single-task (*M* = .83) than in the dual-task (*M* = .54) conditions, *t*(10) = 7.75, *p* < .001, *d* = 2.34 (see Fig. 8a). Again, the accuracy in this central task did not differ significantly between the cued and noncued conditions (*M* = .53, and *M* = .55, respectively), *t*(10) = 1.16, *p* = .274. A repeated measures ANOVA with the factors Cue (cued, noncued) and Task (single, dual) on the detection sensitivities from the object detection task yielded a significant main effect of cue, *F*(1, 10) = 10.85, *p* = .008, *η*_p_^2^ = .52, with higher sensitivity in the cued condition (*M =* 1.09) than in the noncued condition (*M* = 0.85); a significant main effect of task, *F*(1, 10) = 8.37, *p* = .016, *η*_p_^2^ = .46, with higher sensitivity in the single task (*M =* 1.08) than in the dual task (*M* = 0.86); but no significant interaction, *F*(1, 10) = 0.56, *p* = .473, *η*_p_^2^ = .05. These results replicate the findings from Experiment 3a, showing that spatial attention and category-based attention independently enhance object detection in natural scenes (see Fig. 8b).

We next tested whether the metacognitive ability to discriminate between correct and incorrect responses would be differentially affected by spatial and category-based attention. A repeated measures ANOVA with the factors Cue and Task on the mean values of the AUC from the Type II ROC curve yielded neither significant main effects nor an interaction, all *F*s < 1, all *p*s > .622, all *η*_p_^2^s < .03 (Fig. 8c). Additional analyses conducted separately for trials with target-present and trials with target-absent responses showed that metacognitive ability for discriminating between hits and false alarms (trials with target-present responses: AUC *M* = 0.69, *SD* = 0.07) was higher than metacognitive ability for discriminating between correct rejections and misses (trials with target-absent responses: AUC *M* = 0.54, *SD* = 0.03), *t*(10) = 6.29, *p* < .001. A one-sample *t* test showed that participants’ metacognitive ability to discriminate between correct rejections and misses exceeded chance performance, *t*(10) = 5.04, *p* = .001, consistent with the notion of “attentional blindness” in dual-task paradigms (Kanai et al., 2010), in which participants are still able to distinguish their own misses from the actual physical absence of targets. More importantly, however, as with overall metacognitive ability, neither the Cue and Task factors nor their interaction had significant effects on metacognitive ability in either trials with target-present responses, all *F*s < 1, all *p*s > .439, all *η*_p_^2^s < .06, or trials with target-absent responses, all *F*s(1, 10) < 1.68, all *p*s > .223, all *η*_p_^2^s < .15. Thus, consistent with previous findings (Sherman et al., 2015; Wilimzig et al., 2008), the availability of spatial attention did not lead to improved metacognitive performance. Similarly, advance knowledge of the relevant target category did not improve metacognition. This invariance of metacognitive ability to task manipulations is in line with recent research indicating that metacognition may represent a task-independent higher-order cognitive trait that is separable from perceptual performance measures (Song et al., 2011).

#### Experiment 3c

**Fig. 9:**
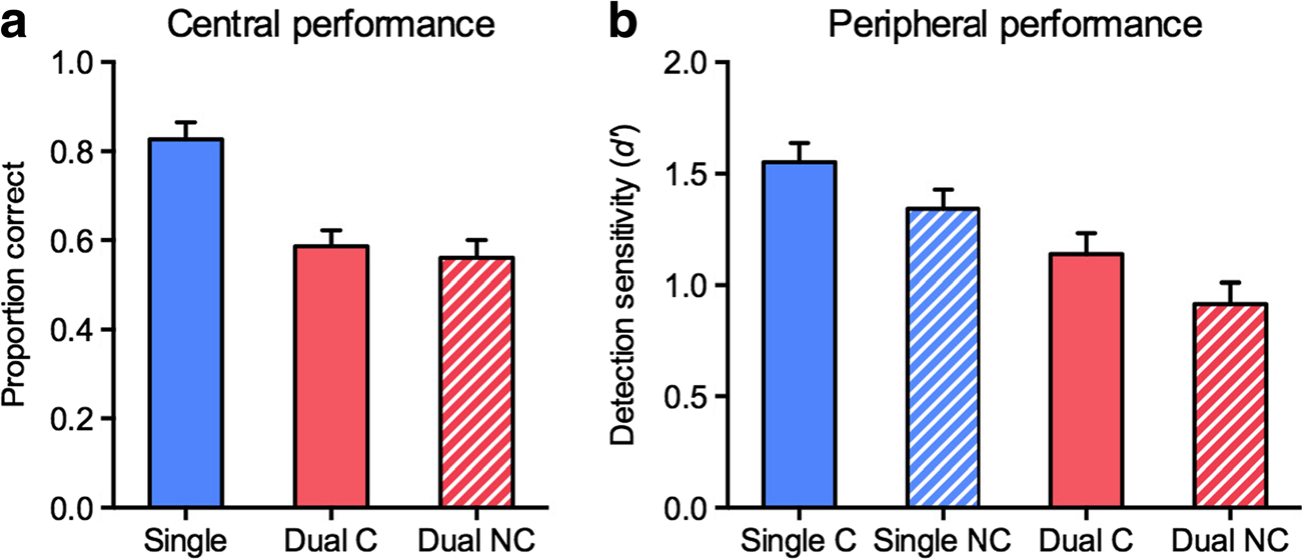
Results from Experiment 3c. (**a**) Mean proportions correct for counting the numbers of central fixation dims, shown separately for the single-task condition, the dual-task cued condition (“Dual C”), and the dual-task noncued condition (“Dual NC”). *Error bars* represent the *SE*s of the differences between the single-task and dual-task conditions. (**b**) Mean *d'* scores for object detection in peripheral scenes, shown separately for the single-task cued (“Single C”), the single-task noncued (“Single NC”), the dual-task cued (“Dual C”), and the dual-task noncued (“Dual NC”) conditions. *Error bars* represent the *SE*s of the differences between the cued and noncued conditions

As in the previous experiments, accuracy in the central fixation-dimming task was higher in the single-task (*M* = .83) than in the dual-task (*M* = .57) conditions, *t*(10) = 6.71, *p* < .001, *d* = 2.02 (see Fig. 9a). In contrast to the previous experiments, in Experiment 3c accuracy in the central task was slightly but significantly higher in the cued dual-task condition (*M* = .59) than in the noncued dual-task condition (*M* = .56), *t*(10) = 2.44, *p* = .035. Note that a difference in this direction in the central dimming task cannot account for any potential benefit in peripheral object detection in the cued relative to the noncued condition. If anything, such a differential trade-off between central and peripheral performance in the cued and noncued conditions would be associated with better performance in the noncued than in the cued condition.

A repeated measures ANOVA with the factors Cue (cued, noncued) and Task (single, dual) on the detection sensitivities from the object detection task yielded a significant main effect of cue, *F*(1, 10) = 12.90, *p* = .005, *η*_p_^2^ = .56, with higher sensitivity in the cued condition (*M =* 1.34) than in the noncued condition (*M* = 1.13); a significant main effect of task, *F*(1, 10) = 21.77, *p* = .001, *η*_p_^2^ = .69, with higher sensitivity in the single task (*M =* 1.45) than in the dual task (*M* = 1.03); but no significant interaction, *F*(1, 10) = 0.01, *p* = .935, *η*_p_^2^ < .01 (see Fig. 9b). Thus, category-based and spatial attention also improved object detection performance with another set of scene photographs, and their beneficial influences were mutually independent.

#### Spatial and category-based attention across experiments

The results from Experiment 3 show that object detection in natural scenes benefits from both category-based and spatial attention, and that there is no interaction between these two types of attention, indicating that they exhibit mutually independent influences. To test for an interaction with more statistical power, we ran an additional ANOVA on peripheral detection performance across these experiments (for the two participants who took part in two of Experiments 3a–3c, the data were averaged across experiments). There were a significant main effect of cue, *F*(1, 33) = 46.34, *p* < .001, *η*_p_^2^ = .58, and a significant main effect of task, *F*(1, 33) = 41.34, *p* < .001, *η*_p_^2^ = .56, but again, no significant interaction, *F*(1, 33) < 0.01, *p* = .963, *η*_p_^2^ < .01. This provides further support that category-based and spatial attention independently influence performance with object detection in natural scenes.

## General discussion

In the present study, we examined the roles and interaction of spatial attention and category-based attention in object detection in natural scenes. First, we found that animal and vehicle detection in natural scenes was better when observers could prepare to detect one specific category than when they had to look for both categories simultaneously. These findings demonstrate the beneficial effect of top-down preparation on detecting superordinate object categories in natural scenes. Second, this influence of category-based attention was independent of spatial attention, improving animal and vehicle detection to similar extents in both single- and dual-task conditions. Third, performance was better in the single-task than in the dual-task condition, even when observers could prepare to detect one specific category, demonstrating that spatial attention improves object detection in natural scenes. Thus, efficient object category in natural scenes requires both category-based and spatial attention.

At the same time, the present study also provides evidence that object perception in real-world scenes is less dependent on spatial attention than is the perception of seemingly simple attributes of more artificial stimuli. When spatial attention was engaged by the central task, color pattern discrimination dropped dramatically, to near-chance-level performance. Consistent with Li et al. (2002), this dual-task cost for color pattern discrimination was much larger than the dual-task cost for object detection in natural scenes. Such efficient perception of natural scenes may reflect the extensive species and individual experience with real-world stimuli. These highly familiar, meaningful stimuli may therefore be “inherently primed” (cf. Lavie, Beck, & Konstantinou, 2014), requiring lower activity and fewer attentional resources to be perceived than simple but artificial stimuli (Greene & Li, 2014; Li et al., 2002; Peelen & Kastner, 2014; Thorpe et al., 1996).

### Spatial attention

In all experiments, object detection in peripheral scenes was improved when spatial attention was available. This impact of spatial attention is seemingly at odds with the results from Li et al. (2002) who found that performance was similar in single- and dual-task conditions. This was interpreted as demonstrating that object categorization in natural scenes can be done in the near absence of attention. How can we account for this apparent discrepancy? First, the two studies differ in their analysis approach: While we conducted group-level statistics involving a total of 34 naïve participants across experiments, Li et al. tested five participants (including two authors) and analyzed performance at an individual-subject level, finding no significant differences between single- and dual-task conditions for individual subjects, with experimental blocks rather than subjects as data points. This approach prevents population-level inferences and is limited in statistical power. Indeed, had they tested more participants using a standard statistical approach, Li et al. might have arrived at a conclusion similar to that of the present study (e.g., see their Fig. 3a–c, showing a drop in object detection accuracy from a single to a dual task in four out of five participants).

Second, it is possible that the central fixation-dimming task used in the present study was simply more taxing on spatial attention than the central search task used by Li et al. (2002). In their study, the finding that central task performance was below ceiling was taken as evidence that spatial attention was fully engaged, leaving virtually no spatial attention for peripheral scene perception. However, as argued by Cohen et al. (2011) below-ceiling performance could reflect noisy sensory signals rather than limited spatial-attentional resources (cf. Norman & Bobrow, 1975). Whereas Li et al. set central-task performance such that it was the same in the single- and dual-task conditions, in the present study and in Cohen et al.’s, central-task performance was below ceiling in the single-task condition, and dropped even further in dual-task conditions. Thus, it is possible that the spatial-attentional requirements of natural scene perception become apparent only with more challenging central tasks (see also Lavie et al., 2014).

### Category-based attention

In addition to spatial attention, the present findings also demonstrate the beneficial influence of category-based attention on object detection in natural scenes. Prior information on the to-be-detected superordinate category improved performance. Previous studies have shown that prior information on basic-level categories and on scene gist can improve perceptual performance in simple detection and scene categorization tasks (Evans et al., 2011; Lupyan & Ward, 2013; Pinto et al., 2015; Stein & Peelen, 2015), and that objects from the cued basic-level object category automatically capture attention (Reeder & Peelen, 2013; Reeder, van Zoest, & Peelen, 2015). The present results show that observers can also prepare for superordinate categories and that such top-down preparation improves perceptual performance. This raises the question for which visual attributes observers prepare when provided with information of the upcoming task-relevant superordinate category (vehicles or animals). For basic-level categories, there is evidence that observers prepare for category-diagnostic object parts such as wheels of a car (Evans & Treisman, 2005; Reeder & Peelen, 2013), and prior information on scene gist may involve preparation for simple visual features and low-level scene statistics (e.g., Groen, Ghebreab, Prins, Lamme, & Scholte, 2013). In the present study, the wide range of different basic-level categories in each superordinate category would have rendered top-down preparation for diagnostic object parts less effective, and the inclusion of incongruent foreground-background scenes precluded preparation for low-level features (e.g., colors). To determine the content of preparatory templates for superordinate categories, future studies may use attentional capture paradigms to test to which stimulus features observers automatically orient while preparing to detect animals or vehicles (Reeder & Peelen, 2013; Reeder et al., 2015).

The beneficial influence of category-based attention is consistent with fMRI, MEG, and TMS evidence for strong top-down influences on neural processing of natural scenes. Patterns of neural responses in object-sensitive cortex to images of natural scenes are dominated by task-relevant objects, while responses to task-irrelevant objects are weaker (Peelen & Kastner, 2011; Peelen et al., 2009) or even suppressed (Seidl, Peelen, & Kastner, 2012). This category-specific modulation of neural activity occurs early in time, modulating the initial categorical representation of the scene (Kaiser, Oosterhof, & Peelen, 2016) and is related to preparatory cue-related activity, prior to the presentation of the scene (Peelen & Kastner 2011; Reeder, Perini, & Peelen, 2015; Soon, Namburi, & Chee, 2013). These preparatory signals may constitute the neural substrate of category-diagnostic templates that bias the processing of scenes in favor of the task-relevant category, providing the neural basis for the beneficial effect of category-based attention observed in the present study. These previous results, together with the present behavioral findings, demonstrate that rather than being a bottom-up driven, automatic process (e.g., Groen et al. 2016) natural scene perception is strongly influenced by the observer’s attentional set.

### Independence of spatial and category-based attention

The influence of category-based attention was independent of spatial attention, improving object detection in both single- and dual-task conditions to a similar extent. This independence from spatial attention is reminiscent of feature-based attention, where attention to simple stimulus features such as orientation, color, or motion enhances processing of these features globally across the visual field, in both spatially attended and unattended locations (e.g., Folk, Leber, & Egeth, 2002; Liu et al. 2007; T. Liu & Mance, 2011; Maunsell & Treue, 2006; Serences & Boynton, 2007). Similarly, search for basic-level object categories (e.g., cars and people) in natural scenes involves spatially global mechanisms, such that objects from the task-relevant category at spatially unattended locations capture attention (Reeder & Peelen, 2013; Reeder et al., 2015) and elicit visual cortex responses (Peelen et al., 2009). The present findings now demonstrate that observers can also prepare for superordinate categories and that this form of category-based attention operates in both spatially attended and unattended locations.

While category-based attention is similar to feature-based attention in its independence from spatial attention, it is unlikely that category-based attention can be reduced to feature-based attention. Rather, to prepare for the combination of visual features that is diagnostic for the presence of animals or vehicles in a natural scene, observers need to set up more complex preparatory templates, representing combinations of multiple low-level features and mid-level shape features, or object parts (Delorme et al., 2010; Evans & Treisman, 2005; Reeder & Peelen, 2013). Through extensive experience these seemingly complex combinations of basic features and shape properties may be processed with similar efficiency as low-level features. Depending on the specific search task, observers may quickly activate such spatially global category-specific “flexible feature sets” (Treisman, 2006), leading to enhanced processing of task-relevant objects in spatially unattended locations. Specifying whether category-based attention indeed relies on the activation of such higher-level, category-specific features or on efficiently conjoining bundles of lower-level basic features represents an important question for future research.

### Conclusion

In conclusion, the present findings confirm previous studies showing that object detection in natural scenes is remarkably more efficient than seemingly simpler tasks on artificial stimuli, with only a relatively small drop in detection performance when spatial attention is unavailable. This efficient perception of target objects in natural scenes reflects the efficient detection of diagnostic target features rather than the detection of global features of the background scene. Importantly, the present findings also show that efficient detection in natural scenes requires attention, since both spatial attention and nonspatial (category-based) attention improved detection performance. We conclude that efficient natural scene perception critically depends on the attentional state of the visual system prior to scene onset.
